# Mosquito Gut Microbiota: A Review

**DOI:** 10.3390/pathogens13080691

**Published:** 2024-08-15

**Authors:** Hongmei Liu, Jianhai Yin, Xiaodan Huang, Chuanhui Zang, Ye Zhang, Jianping Cao, Maoqing Gong

**Affiliations:** 1 Key Laboratory of Parasite and Vector Biology, National Health Commission of People’s Republic of China, National Institute of Parasitic Diseases at Chinese Center for Disease Control and Prevention (Chinese Center for Tropical Diseases Research), Shanghai 200025, China; yinjh@nipd.chinacdc.cn; 2 Digestive Disease Hospital of Shandong First Medical University, Shandong Institute of Parasitic Diseases, Shandong First Medical University & Shandong Academy of Medical Sciences, Jining 272000, China; jysywb@126.com (X.H.); zch18853812382@163.com (C.Z.); zyxjja@163.com (Y.Z.); 3 World Health Organization Collaborating Centre for Tropical Diseases, Shanghai 200025, China

**Keywords:** gut microbiota, insecticide resistance, mosquito, environment

## Abstract

Mosquitoes are vectors of many important human diseases. The prolonged and widespread use of insecticides has led to the development of mosquito resistance to these insecticides. The gut microbiota is considered the master of host development and physiology; it influences mosquito biology, disease pathogen transmission, and resistance to insecticides. Understanding the role and mechanisms of mosquito gut microbiota in mosquito insecticide resistance is useful for developing new strategies for tackling mosquito insecticide resistance. We searched online databases, including PubMed, MEDLINE, SciELO, Web of Science, and the Chinese Science Citation Database. We searched all terms, including microbiota and mosquitoes, or any specific genera or species of mosquitoes. We reviewed the relationships between microbiota and mosquito growth, development, survival, reproduction, and disease pathogen transmission, as well as the interactions between microbiota and mosquito insecticide resistance. Overall, 429 studies were included in this review after filtering 8139 search results. Mosquito gut microbiota show a complex community structure with rich species diversity, dynamic changes in the species composition over time (season) and across space (environmental setting), and variation among mosquito species and mosquito developmental stages (larval vs. adult). The community composition of the microbiota plays profound roles in mosquito development, survival, and reproduction. There was a reciprocal interaction between the mosquito midgut microbiota and virus infection in mosquitoes. *Wolbachia*, *Asaia*, and *Serratia* are the three most studied bacteria that influence disease pathogen transmission. The insecticide resistance or exposure led to the enrichment or reduction in certain microorganisms in the resistant mosquitoes while enhancing the abundance of other microorganisms in insect-susceptible mosquitoes, and they involved many different species/genera/families of microorganisms. Conversely, microbiota can promote insecticide resistance in their hosts by isolating and degrading insecticidal compounds or altering the expression of host genes and metabolic detoxification enzymes. Currently, knowledge is scarce about the community structure of mosquito gut microbiota and its functionality in relation to mosquito pathogen transmission and insecticide resistance. The new multi-omics techniques should be adopted to find the links among environment, mosquito, and host and bring mosquito microbiota studies to the next level.

## 1. Introduction

Microbiotas, including bacteria, archaea, protists, fungi, and viruses, are a range of microorganisms that may be commensal, mutualistic, or pathogenic and are found in and on other organisms. Human microbiome study started in the 17th century [1]. The human gut microbiota, especially its relationship with human health, is probably the best studied microbiota among all living organisms [1,2,3]. Bacteria are the largest and, to date, the most studied component of the human microbiota [4], as supported by the development of many types of antibiotics [5]. The gut microbiota is considered the master of host development and physiology [6,7,8]. The gut microbiota affects nutrient absorption, immunity development, direct defense against pathogens, and many other roles [9,10,11,12,13]. In the view of drug metabolism mediated by the gut microbiota, it includes the modification of the chemical structure of drugs by microbial metabolite enzymes and/or affecting the expression of host metabolizing enzymes such as cytochrome P450 [14]. These studies are parallel to the discovery of the metabolizing enzyme cytochrome P450 in insects, especially agricultural pests and human disease vectors such as mosquitoes [15,16,17], promoting the study of the mechanism of insecticide/pesticide resistance in insects. Nonetheless, research in microbiota is an area with rapid development, with the new techniques of multi-omics (genomics, metagenomics, proteomics, transcriptomics, metabolomics, and fluxomics) at the forefront [18]. 

In addition to humans and other vertebrates, insects also have complex and diverse gut microbiota [19,20,21,22]. Insect gut bacterial diversity is determined by environmental habitat, diet, developmental stage, and phylogeny of the host [23,24,25]. The gut microbiota of insects not only contribute to nutrition, protection from parasites and pathogens, modulation of immune responses, and communication [22], but also affect insect growth, development, survival, and fitness [22,26]. Particularly, there is a strong link between insect gut microbiota and insecticide resistance [27,28,29,30,31,32], mainly through insecticide degradation [32,33,34] and metabolic detoxification [31,35,36,37,38]. 

The mosquito (Culicidae family) gut microbiota study has increasingly gained attention [38,39,40]. Mosquitoes are vectors of many human and zoonotic diseases, including *Anopheles*, which transmits malaria parasites (the deadliest of all tropical infectious diseases); *Aedes*, which transmits dengue virus (DENV), Chikungunya virus (CHIKV), yellow fever virus, and Zika virus (ZIKV); and *Culex*, which transmits West Nile virus (WNV), filarial parasites, and different kinds of human and zoonotic encephalitis viruses (e.g., Japanese encephalitis virus, eastern equine encephalitis virus, La Crosse encephalitis virus, St. Louis encephalitis virus) [41,42,43]. Currently, no effective treatment or vaccine is available for many diseases caused by these pathogens, such as dengue and Chikungunya fevers, except for a small number of diseases such as malaria (drug treatment) and yellow fever (vaccine), leaving vector controls as the primary option for prophylaxis. Recently, many studies have linked these infections and diseases to the mosquito gut microbiota [39,40,44,45,46,47,48]. However, the interaction between disease transmission and mosquito gut microbiota is dependent on mosquito species and specific diseases. For example, in *Aedes* mosquitoes, most of the focus was on the preventive effect of the bacteria genus *Wolbachia* on *Aedes*-borne diseases such as dengue fever and Zika [47,49,50]. In WNV-infected *Culex* mosquitoes, the results were variable regarding the directionality of this relationship, although these studies suggested that bacteria of the genera *Serratia* and *Enterobacter* contribute to WNV development [39]. Studies of *Culex* mosquitoes in Asia show complex interactions between Japanese encephalitis virus and other virus infections in the mosquito gut [51,52]. However, studies on *Plasmodium*-*Anopheles* gut microbiota interactions are relatively limited in the literature [53], although it was reported that the microbiota of both the mosquito and the human host play important roles in *Plasmodium* parasite transmission, malaria progression, and clearance of *Plasmodium* infection [54].

Insecticides have been widely used since the 1950s in the form of indoor residual spraying (IRS) [55], ultra-low volume (ULV) outdoor spray [56], aerial spraying [57], and insecticide-treated nets [58], not only for mosquito control for public health, but also for agricultural pest control, which leads to the development of extensive resistance to all five classes of insecticides (pyrethroid, organochloride, carbamate, phosphorothioate, and pyrrole) in mosquitoes [17,59]. Mechanisms of mosquito insecticide resistance include knockdown resistance (*kdr*) caused by target site gene mutations [60], metabolic resistance caused by the elevated expression of esterases, monooxygenases P450 and glutathione S-transferases enzymes [61,62,63,64,65], and reduced insecticide penetration due to cuticle thickening and modification [66]. In addition, multiple resistance has become increasingly common in different mosquitoes [67,68]. Currently, the potential link between insect gut microbiota and insecticide resistance has been gradually revealed [16,69,70,71,72,73,74], especially host detoxification ability, which is currently the point of interest [16,35,75,76,77].

The aim of this study is to review mosquito gut microbiota, with special emphasis on the role of gut microbiota in preventing disease transmission and the interplay with insecticide resistance. The implication of mosquito microbiota in disease control has been reviewed previously [18,38], therefore it was not included in this review. In addition, this review focused on mosquito-transmitted pathogens that cause human diseases rather than zoonotic diseases.

## 2. Methods

### 2.1. Protocols

For this review, mosquito microbiota are defined as studies that involve both mosquitos and microbiota. General studies of individual microorganisms, such as molecular detections of an individual microorganism (for example, *Wolbachia*), were mentioned in this study, but they were either summarized in a special way (see the “Study Selection 2.4” below) or not considered in the general summary. However, studies to confirm the functionality of gut microorganisms were considered in this review. Field studies of *Wolbachia* as a disease control agency were excluded from this review.

The primary focuses of this review are (1) the interactions among the environment, mosquito microbiota, and disease transmission; (2) the key microorganisms that reside in mosquito bodies that are important for disease or vector control; and (3) the interactions between the microbiota and mosquito insecticide resistance.

### 2.2. Search Strategy

The search terms consisted of the 11 searches specified below. Although some of the searches overlap, we found that all were necessary.

(Mosquito microbiota) or (mosquito microbiome);
(*Aedes aegypti* microbiota);
(*Aedes albopictus* microbiota);
(*Culex* microbiota);
(*Anopheles* microbiota);
(*Aedes* microbiota);
(*Armigeres* microbiota);
(*Haemagogus* microbiota);

The following searches for special bacteria are based on a preliminary summary of published studies, which shows that these commonly occurring bacteria affect the development, survival, and insecticide resistance of mosquitoes, and pathogen infections in mosquitoes.

(Mosquito *Wolbachia*);
(Mosquito *Asaia*);
(Mosquito *Serratia*).

*Wolbachia*, *Asaia*, and *Serratia* are in fact the three most studied bacteria associated with the three classes of the deadliest mosquito-borne human pathogens, i.e., *Aedes*-borne dengue, Chikungunya, and Zika viruses, *Anopheles*-borne *Plasmodium* malaria parasites, and *Culex*-borne encephalitis virus (both human and zoonotic).

Databases searched include PubMed, MEDLINE, SciELO, Web of Science, and Chinese Science Citation Database (CSCD). SciELO focuses on Spanish- and Portuguese-language journals. CSCD includes all journals hosted by Chinese institutions. The date of the final search was 31 July 2023.

### 2.3. Study Eligibility Criteria

**Figure 1:**
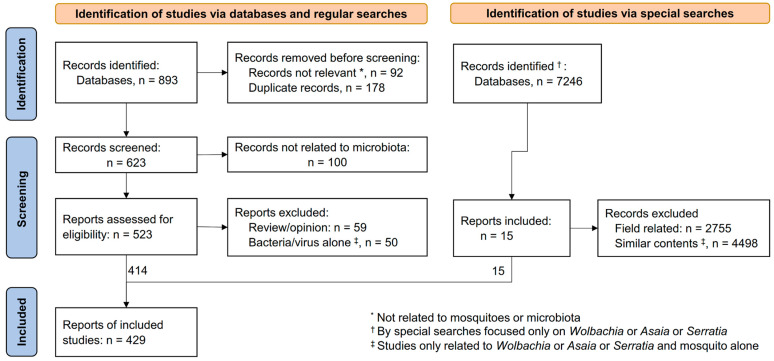
Flowchart of the article search and screening process.

All studies involving microbiota and mosquitoes were included in the initial selection (Figure 1), including both larval and adult mosquitoes of any species. Studies including the following experiments were included: (1) general exploration of microbiota in mosquito larvae and adults; (2) the effect of microbiota on mosquito development, survival, and reproduction; (3) the effect of microbiota on pathogen transmission; and (4) the interactions between mosquito microbiota and mosquito larval or adult resistance to insecticides. However, pure semi-field or field experiments or trials involving the assessment of intervention efficacy or effectiveness under natural conditions, for example, field work evaluating the effectiveness of *Wolbachia* infection on *Aedes* population reduction, were included in the general review but not included in the final study selection.

Inclusion criteria:

Mosquito larval and adult laboratory experiments or analysis of the community structure of the microbiota;

Mosquito larval and adult laboratory experiments to confirm the functionality of specific microorganisms isolated from mosquito microbiota;

Mosquito larval and adult laboratory experiments that compare differences in microbiota community structures between mosquitoes collected from different sites or between mosquito species;

Mosquito larval and adult laboratory experiments or molecular analyses that study the interactions between mosquito insecticide resistance and microbiota or microorganisms isolated from mosquito microbiota.

Exclusion criteria:

Pure field or semi-field experiments under natural or semi-natural conditions to evaluate the efficacy or effectiveness of *Wolbachia* or other microorganisms in controlling mosquito populations or pathogen transmission;

Studies that purely focus on methodology development, pathogen or disease transmission, isolation, or confirmation of the existence of a single microorganism;

Studies that were conducted under unconventional conditions or with unconventional standards.

### 2.4. Study Selection

Studies were selected manually by checking their results, with exceptions for pure *Wolbachia*, *Asaia*, and *Serratia* studies (Figure 1). If a study included the words microbiota and mosquito in the title and abstract, the study was included in this review for further analysis. Data generated from studies under unconventional conditions or with unconventional standards were excluded from this review. For the special bacteria *Wolbachia*, *Asaia*, and *Serratia* and mosquito interactions, such as how these bacteria affect mosquito infection of pathogens (e.g., *Wolbachia* preventing dengue virus infection in *Ae. aegypti*), only up to five published articles for each bacteria were included in this review since most of these studies used similar methods and yielded very similar results.

The data were manually extracted. The outcomes include interactions between microbiota and mosquito development, survival, pathogen infection, and insecticide resistance. Although qualitatively summarized results were presented in this review, we did not conduct meta-analyses, especially quantitative summaries of published studies, as a broad range of results was presented in the results.

## 3. Results

### 3.1. Description of Search Results

The multiple combined search terms yielded a total of 893 records from regular searches and 7246 from special searches. After the removal of duplicates (*n* = 178) and articles with non-relevant contents (*n* = 92), 623 records from the regular search were deemed eligible for full-text screening (Figure 1). Among these, 100 records were not related to microbiota. Additional records were removed, including 59 review or opinion articles that are not closely related to microbiota and mosquitoes and 50 focused on studies of individual bacteria/viruses alone with mosquitoes (Figure 1). For the special search, only 15 articles were included in the final review. Overall, 429 studies were included in this review (Figure 1).

### 3.2. Mosquito Gut Microbiota: Environment, Disease Transmission, and Mosquito Development

The mosquito gut microbiota shows a complex community structure with rich species diversity, and dynamic changes in the species composition over time and across space [78,79,80,81,82,83]. For example, 18 phyla, 138 families, and 337 genera of microorganisms were identified from the larval gut of *Anopheles gambiae* sensu lato in naturally collected samples in Kenya, while only 42 families of microorganisms were found in the gut of a 3-day-old sugar-fed adult [79]. Meanwhile, the larval gut bacterial microbiota (dominant family *Chlorophyta*, 17%) has a similar structure as that in its habitat (dominant family *Chlorophyta*, 16%), while the diversity reduced as the larvae developed into pupa (dominant family *Aeromonas*, 31%) [79]. Furthermore, gut bacterial diversity and dominant species changed dramatically in adult mosquitos after sugar feeding (7-day-old, dominant family *Elizabethkingia*, 62%) and blood feeding (7-day-old, dominant family *Elizabethkingia*, 84%), indicating a lowered diversity [79]. A similar dynamic in gut microbiota community structure along with the growth stages was also reported in *Aedes albopictus* [78]. Interestingly, in a laboratory strain of *Ae. albopictus* fed with brewer’s yeast *Saccharomyces cerevisiae* cells, *Wolbachia* accounted for a tiny proportion of bacterial reads at the larval stage (7% at day 3), but it reached 80% of the total bacterial reads in 17-day-old adults [78]. For *Culex* mosquitoes, although the species of microorganisms and the community structure in the gut microbiota are very different from the above two mosquitoes, a general decreasing trend in the number of observed species and diversity was also observed over time [84]. In addition, substantial change was found in the diversity and relative abundance of different families (species, genera) of microbiota over seasons, regardless of mosquito species [84,85].

The spatial heterogeneity in the mosquito larval gut microbiota community is likely associated with their living environment. For example, significant differences were found in the gut microbial composition among different species of field-collected mosquitoes (*Ae. albopictus*, *Aedes galloisi*, *Culex pallidothorax*, *Culex pipiens*, *Culex gelidus*, and *Armigeres subalbatus*) at the same collection location and substantial variations in the gut microbiota among individuals of the same mosquito species collected at different geographical locations (eight sites in Hainan, China) [80]. However, similar bacterial diversity and evenness were found among mosquito species across the four genera, indicating that the mosquito midgut plays an important role in regulating the colonization and assembly of bacterial communities [80,86,87].

The microbiota has also been implicated in pathogen infections in mosquito vectors. It was reported that there was a complex microbiota community structure in the midgut of *Aedes aegypti* mosquitoes, in which there was a reciprocal interaction between the mosquito midgut microbiota and dengue virus infection, i.e., a marked decrease in susceptibility to dengue virus infection was found when *Ae. aegypti* was infected with the bacteria species *Proteus* sp. and *Paenibacillus* sp.; conversely, the dengue virus infection influenced the microbial load in the mosquito midgut [88]. In contrast, gut infection with *Serratia marcescens* in *Ae. aegypti* could facilitate dengue virus infection [89]. Moreover, *Anopheles stephensi* mosquitoes were more susceptible to *Plasmodium berghei* infection when their native microbiota was cleared with antibiotics compared to the untreated mosquitoes [90], indicating that bacteria in the midgut may help to prevent *P. berghei* infection. Similarly, a strong correlation between the abundance of bacterial *Enterobacteriaceae* and *Plasmodium falciparum* infection was found in *An. gambiae* mosquitoes [86]. In fact, *P. falciparum* development in the mosquito gut was affected by many bacterial isolates (e.g., *Escherichia coli*, *Serratia marcescens*, and *Pseudomonas stutzeri*) in challenge experiments [91].

Mosquito gut microbiota play profound roles in mosquito growth and development, survival, and reproduction [92,93,94,95]. For example, the *Ae. aegypti* mosquito’s survivorship and fecundity were reduced after midgut microbial clearance using antibiotic treatment [45]. Similarly, adult *Anopheles arabiensis* mosquito longevity could be increased after their midgut was treated with the broad-spectrum bactericidal antibiotic gentamycin, the Gram-positive narrow-spectrum antibiotic vancomycin, and the Gram-negative narrow-spectrum antibiotic streptomycin, while longevity was significantly reduced under treatment with the broad-spectrum bacteriostatic antibiotic erythromycins [93], indicating the differential influence of different bacteria in the mosquito gut and their complex balance in supporting mosquito survival. Furthermore, *An. stepehsni* adult longevity, fecundity, and infection with malaria parasites would be reduced after their midguts were treated with tetracycline [96]. Unfortunately, the exact microorganisms responsible for these changes were unknown in the above studies. *Bacillus* and *Staphylococcus* in the midgut were tested to be essential for the normal and high fecundity of *Culex pipiens* [97], and *Wolbachia* (*wPip*) could contribute to the higher longevity and improved survivorship of *Culex quinquefasciatus*, but with lower reproductive fitness [98,99]. In addition, the antibiotic-treated *An. gambiae* adult mosquitoes had a reduced lifespan with a median survival of 14 days, while the untreated mosquitoes survived up to a median of 22 days, whereas an increase in egg hatchability and larval development were observed after the reintroduction of *Enterobacter cloacae* and *Serratia marcescens* bacteria in the mosquito [100].

### 3.3. Key Bacteria in Mosquito Gut Microbiota: Importance for Preventing Disease Transmission

As illustrated earlier, bacteria are the most studied component of the gut microbiota. Being part of the microbiota community, bacterial symbionts are abundant in the mosquito gut (both larval and adult) and influence mosquito development, survival, and reproduction [83,93,96,101,102]. The community structure of bacterial endosymbionts has been examined in many mosquito species, including *Ae. aegypti* [103,104,105], *Ae. albopictus* [104,105], *Ae. galloisi* [80], *Aedes triseriatus* [106,107], *Aedes japonicus* [107,108], *An. gambiae s.l.* [104,109,110,111,112], *Anopheles funestus* [109,110], *An. stephensi* [104,113], *Ar. subalbatus* [80], *Cx. gelidus* [80,114], *Cx. pallidothorax* [80], *Cx. pipiens* [80,106,108,115], *Cx. quinquefasciatus* [116], *Culex restuans* [115], *Culex tritaeniorhynchus* [114], *Mansonia annulifera* [114], and *Psorophora columbiae* [106]. The bacterial community from mosquito gut is usually dominated by a few (4–5) genera regardless of mosquito species, although many species of bacteria have been found from mosquito gut [108,112,116]. For example, the most abundant genera were *Enterobacter* (32.8%) and *Aeromonas* (29.8%), followed by *Pseudomonas* (11.8%), *Acinetobacter* (5.9%), and *Thorsellia* (2.2%) [112] identified from the gut of *An. gambiae*. Similarly, four bacterial genera were the most abundant in gut from *Cx. pipiens* and *Ae. japonicus*, with the most prevalent genera being *Sphingomonas* and *Rahnella*, respectively [108]. Here, we focused the review on the three bacteria that may interfere with the transmission of these three pathogens.

The most well-known mosquito endosymbiont bacterial genus is probably the intracellular bacteria of *Wolbachia*, which infects a high proportion of insects and is possibly the most common reproductive parasite in the biosphere [117,118,119]. It has been commonly detected in different species of mosquitoes, including natural populations of *Aedes*, *Culex*, *Mansonia*, and *Armigeres* mosquitoes all over the world [120,121,122,123], and it is the predominant bacterial genus in many *Aedes* and *Culex* mosquitoes [78,80,115,124,125], but natural infection of *Wolbachia* in *Anopheles* mosquitoes is uncommon [126,127,128]. *Wolbachia* is of special interest due to its unique biological and pathogenetic characteristics that induce reproductive manipulation phenotypes, including parthenogenesis, feminization, cytoplasmic incompatibility, and male-killing, which increase the endosymbiont’s reproductive success [121,122,123,129]. Thus, it acts as a biocontrol agent against arbovirus vectors [130,131,132]. Although only *Wolbachia*-infected females can pass the infection on to their offspring, *Wolbachia* bacteria maximize their transmission by significantly altering the reproductive capacity of their hosts through male killing [133], feminization [134], parthenogenesis [135], and cytoplasmic incompatibility, respectively [125,126,136].

Biological control using *Wolbachia* is based on two management strategies, i.e., population suppression using the incompatible insect technique (IIT) and population replacement using anti-virus mosquito strains. Cytoplasmic incompatibility has been proposed as a tool to suppress mosquito populations and decrease the arbovirus burden in humans [132,137,138,139]. Population suppression occurs when males infected with *Wolbachia* are released into the environment to reproduce with *Wolbachia*-free females, which leads to cytoplasmic incompatibility between gametes and thus cannot produce viable offspring [49,50,140]. The continued release of infected males over time reduces the target mosquito population at a given site [141,142]. This strategy has been effectively used in different countries for *Aedes* mosquito control [141,142,143]. In the replacement strategy, females infected with a specific strain of *Wolbachia* can reduce the replication of arthropod-borne viruses (arboviruses) such as DENV, ZIKV, CHIKV, and WNV [49,50,99,144].

Secondly, *Asaia* is a versatile acetic acid bacterial symbiont capable of cross-colonizing insects of phylogenetically distant genera and orders of agricultural insects and mosquitoes [145,146,147]. It is commonly found in *Anopheles* mosquitoes, including *An. gambuae*, *An. stephensi*, and *An. funestus*, as well as *Monsonia* mosquitoes, and is the dominant bacterial symbiont [148,149,150]. For example, it was found that the bacterial genus *Asaia* predominated the adult internal and cuticle surface microbiota in *Anopheles albimanus*, making up at least 70% of the taxa in each microbial niche across all collection sites [66]. Moreover, *Asaia* has also been detected in *Aedes*, *Culex*, and other mosquito species [124,147,148]. Interestingly, co-infection of *Wolbachia* and *Asaia* in mosquitoes is uncommon, although it has been detected in uncommon *Aedes* mosquito species other than *Ae. aegypti* and *Ae. albopictus* [147,151], and in some other mosquitoes such as *Culex* and *Haemagogus* species [124,151,152]. Meanwhile, the abundance of *Asaia* is usually very low in these co-infected mosquitoes compared to the accompanied mosquitoes [124,147,151]. In fact, it was reported that the presence of one of the symbionts could prevent the establishment of the second one in some mosquito species, and there were microbial niche differences between *Wolbachia* and *Asaia*, i.e., a reciprocal negative interference in terms of the colonization of the gonads [104]. The mutual exclusion or competition between *Asaia* and *Wolbachia* may help explain the inability of *Wolbachia* to colonize the female reproductive organs of *Anopheles* mosquitoes, inhibit its vertical transmission, and explain the absence of *Wolbachia* infection in *Ae. aegypti* and in the majority of natural populations of *Anopheles* mosquitoes [104]. Furthermore, this co-exclusion pattern between *Wolbachia* and *Asaia* was also found in *Ae. albopictus* and *Cx. quinquefasciatus* naturally, and *Asaia* is able to colonize reproductive organs and salivary glands in species uninfected with *Wolbachia* such as *An. gambiae*, *An. Stephensi*, and *Ae. aegypti* [81,104,151,153]. More importantly, it was found that the infection of *Asaia* in *Anopheles* mosquiotes could inhibit *Plasmodium* infections [154,155], which renders its potential as a paratransgenic weapon against malaria [156,157].

Thirdly, *Serratia* is another important genus of mosquito gut bacteria, which is a genus of Gram-negative, facultatively anaerobic, rod-shaped bacteria of the family *Enterobacteriaceae*. It has been found in many mosquito species, including *Ae. aegypti*, *Culex*, *Anopheles*, and *Armigeres* mosquitoes, and has been linked to mosquito development and survival, insecticide resistance, and malaria infections [80,100,150,158,159,160]. Moreover, it can be genetically engineered to prevent malaria parasites in mosquitoes [161,162].

### 3.4. Mosquito Gut Microbiota Mediating Insecticide Resistance

The impacts of insecticides on mosquito microbiota have been studied on most mosquitoes, including *Ae. aegypti* [47,73,75,163], *Ae. albopictus* [164,165], *An. albimanus* [166], *An. arabiensis* [93], *An. coluzzi* [150], *Anopheles gambiae sensu stricto* [149], *An. stephensi* [28], *Cx. pipiens* [167], and *Cx. quinquefasciatus* [74] (Table 1). Most focused on adult mosquitoes and chemical insecticides, with a lesser focus on larval and microbial larvicide *Bacillus thuringiensis* var. *israelensis* (*Bti*). In all cases, insecticide resistance or exposure leads to the enrichment or reduction in certain microorganisms in resistant mosquitoes while enhancing the abundance of other microorganisms in insect-susceptible mosquitoes, and they involve many different species/genera/families of microorganisms [28,74,168] (Table 1). For example, the genera *Coprococcus* and *Ruminococcus* (class *Clostridia*) [169], *Bilophila* (class *Deltaproteobacteria*), *Enterobacter* (class *Gammaproteobacteria*), *Porphyromonas* (class *Bacteroidia*), *Bifidobacterium* (class *Actinobacteria*) [168], *Weissella* (class *Bacilli*), and *Delftia* (class *Betaproteobacteria*) were enriched in the resistant group of *Ae. aegypti* [47] (Table 1). Whereas the species of *Bacteroides faecichinchillae* was significantly decreased in the midguts of resistant *Ae. aegypti* and *Akkermansia muciniphila* was increased in insecticide-susceptible mosquitoes [47,163] (Table 1). However, resistance development is usually accompanied by reduced diversity in microorganisms [69]. In addition, these changes in microbiota community structure and predominant species or genera of microorganisms vary among mosquito species and among different studies of the same species at different sites. For example, *An. albimanus* resistant to fenitrothion, an organophosphorus insecticide, enriched the abundance of *Bacillus* and *Klebsiella pneumoniae*, while the resistance to pyrethroids increased the abundance of *Pseudomonas fragi* and *Pantoea agglomerans* [69,166,167] (Table 1).

Conversely, there are effects of gut microbiota on insecticide resistance. Microbiota can promote insecticide resistance in their hosts by isolating and degrading insecticidal compounds or altering the expression of host genes [31]. Due to the tedious nature of the infection process, only the bacteria *Wolbachia*, *Streptococcus pyrogenes*, *Escherichia coli*, *Serratia oryzae*, and *Acinetobacter junii* [93,164,165,170,171,172], and the fungi *Metarhizium anisopliae* or *Beauveria bassiana* [173,174,175] have been used in the microorganism challenge studies [173,174,175,176] (Table 2), but the results are inconsistent. Scathes et al. found that adding cultured gut bacteria isolated from mosquito larvae to antibiotic-cleared larval food can significantly reduce *Ae. aegypti* larval mortality against propoxur and naled larvicides [177]. Wang et al. further found that the survival of *Serratia oryzae*-enriched *Ae. albopictus* mosquitos significantly increased under deltamethrin, and metabolic detoxification enzymes esterase, P450, and GST were also increased in *S. oryzae*-enriched mosquitoes [165]. Meanwhile, carboxylesterase activity was detected in *S. oryzae*, which can degrade deltamethrin in vitro, and the degradation efficiency was positively correlated with time and bacterial amount [165] (Table 2). Inversely, it was found that supplementation of the bacteria *Streptococcus pyrogenes* or *Escherichia coli* could increase insecticide tolerance to the insecticides deltamethrin and malathion but decrease tolerance under antibiotic treatment via sugar feeding [93]. However, it was found that α-esterase activity was decreased in both resistant and susceptible females after antibiotic (gentamicin, streptomycin, and vancomycin) or bacteria enrichment (heat-killed *S. pyrogenes* or live *E. coli*) treatment, while GST and P450 enzyme activities remained unchanged [93] (Table 2). These unusual results may be caused by other resistance mechanisms. More controversial is the case of fungi *Metarhizium anisopliae* or *Beauveria bassiana* [93,173,174,175,178,179]. Clearly, mosquitoes will be killed by infection with both fungi, regardless of insecticide resistance levels [175,179]. In fact, *Me. anisopliae* infection can lead to very high (up to 97%) mortality in *Ae. aegypti*, *An. Stephensi*, and *Cx. quinquefasciatus* larvae [176,179]. On the other hand, the activities of acetylcholinesterase (AChE), glutathione S-transferase (GST), esterase (EST), acid phosphatases (ACP), and alkaline phosphatases (ALP) were increased in the chlorpyrifos-selected *Cx. quinquefasciatus* mosquitoes but suppressed when exposed to *Me. anisopliae* or *Be. bassiana* [173] (Table 2). Therefore, whether the anti-mosquito effects of the combination of these fungi and insecticides are additive or synergistic warrants further investigation.

In addition, the species composition of the gut microbiota has been linked to insect susceptibility to insecticides [31,180]. Although many insecticide-degrading microorganisms have been reported from soil samples or agricultural insects [18,35,76,181,182,183], few species have been reported from mosquitoes [69] (Table 3). We listed some insecticide-degrading microorganisms found in mosquito guts in Table 3; however, the function was only confirmed in one study [69].

### 3.5. Perspectives

**Figure 2:**
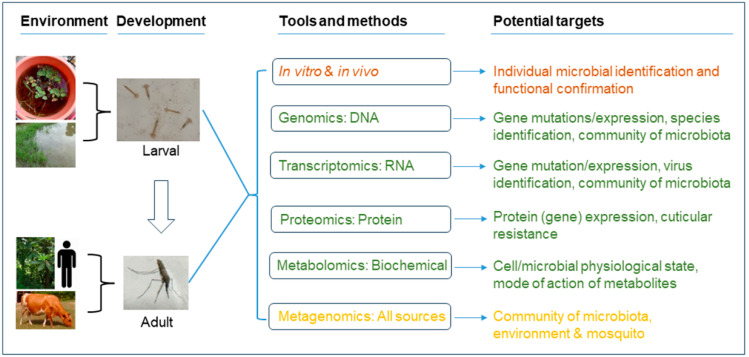
Environments involved in mosquito development and tools and methods for the analysis of mosquito microbiota.

So far, most mosquito gut microbiota studies are more descriptive and exploratory and lack in-depth analysis. Future studies on the mosquito gut microbiota and its functions should better utilize advanced techniques such as state-of-the-art multi-omics tools [18] (Figure 2). Exploring the community ecology of mosquito gut microbiota is necessary; however, we need to further investigate the function of the community structure or the function of the major symbiont microorganisms, and the environment—mosquito gut microbiota—host interactions. These studies can target DNA, RNA, proteins, biochemicals, and complex mixing levels (Figure 2). At the core of all studies is in vitro and in vivo laboratory identification and functional confirmation (Figure 2). Genomics (DNA) and transcriptomics (RNA) can be used to identify gut microbiota community composition and structure; proteomics can be used to examine protein expression levels; metabolomics can be used to assess the metabolites associated with certain functions, such as P450 enzyme levels versus insecticide resistance intensity; and metagenomics can be used to investigate the genetics of multiple sources of samples [18]. Most of these techniques have been used in different studies but not in an integral minor [18,31,184].

**Figure 3:**
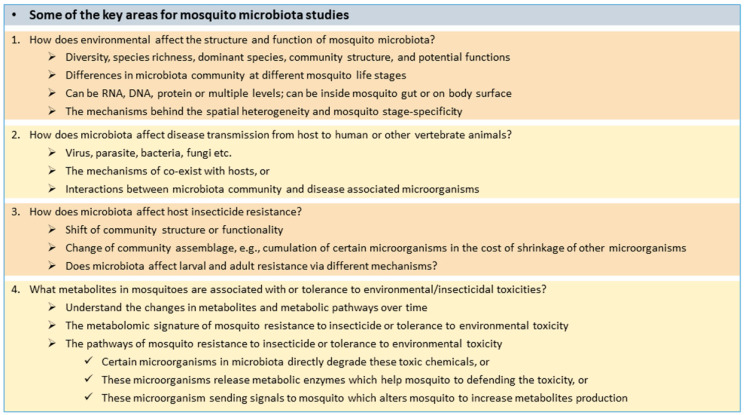
Some of the key areas for mosquito microbiota studies.

There are many unsolved issues in environment–microbiota–mosquito (host) interactions. We proposed four major areas for future studies of the mosquito microbiota (Figure 3). Some of these questions can be answered directly. For example, differences in the microbiota community at different mosquito stages can be solved through simple DNA/RNA sequencing (Figure 3, Question 1). However, what causes these differences may need further in vitro or in vivo experiments to confirm. Why do disease agents such as viruses co-exist with mosquitoes but cause human disease (Figure 3, Question 2)? Do disease-agent-carrying mosquitoes have different microbiota communities than disease-agent-free mosquitoes? In other words, does the microbiota support mosquitos coexisting with these disease agents? Studies have found that mosquito larvae and adults have different levels of tolerance to the same insecticide [185,186]. Does this have anything to do mosquito microbiota community structure (Figure 3, Question 3)? Mosquito gut microbial clearance and reintroduction studies find that the existence of gut microbiota affects mosquito metabolic enzyme levels. What is the pathway for this effect (Figure 3, Question 4)? We know that some bacteria can produce metabolic enzymes [187]. Does the mosquito microbiota directly release microbial enzymes to metabolize insecticides? Or do microorganisms influence the host receptors and signaling pathways through microbial metabolites (Figure 3, Question 4)? Many of these questions may be answered by the integration of multiple techniques; this may be the most robust way to move science forward.

### 3.6. Concluding Remarks

Compared to the extensive research that has been accomplished with microbiota in agricultural insects [76,183], research in mosquito microbiota needs a major catch-up. Although microbiota certainly affect mosquito resistance to insecticides, evidence is scarce after reviewing the existing literature. Insecticide selections enrich certain microorganisms in mosquito microbiota, but the specific microorganisms selected depend on the type of insecticides and mosquito species. Based on experiments involving the removal/addition of individual microorganisms, it seems that insecticide resistance in mosquitoes is likely supported by a combination of multiple microorganisms. Here are some of the areas that need in-depth investigations, e.g., diversity of symbiotic microbiota and their role in mosquito physiology and ecology, functions of mosquito gut microbiota at either individual microorganism or group/community levels, molecular mechanisms of insecticide resistance by insect gut microbiota using multi-omics approaches, the role of gut microbiota in the biodegradation of insecticides, and microbial metabolic pathways related to insecticide resistance. Mosquitoes may go through different physiological and/or genetic changes, such as gene mutations, to defend against insecticides; meanwhile, changes in mosquito microbiota, either actively or passively, may contribute to the mosquito’s defense system in a complementary or supplementary way. The extent of these roles is still unclear and awaits further study. Understanding the relationships between mosquito gut microbiota and mosquito insecticide resistance and the mechanisms by which the microbiota affects mosquito insecticide resistance is useful for developing new strategies for tackling insecticide resistance.

## Figures and Tables

**Table 1:** Impact of insecticide resistance on microbiota.

Mosquito Species	Insecticide	Mosquito Stage	Description of Findings	Reference
*Ae. aegypti*	Permethrin selection	Adult	*Cutibacterium*, *Corynebacterium*, *Citricoccus*, *Leucobacter*, *Acinetobacter*, *Dietzia*, and *Anaerococcus* spp. were more abundant in the selected strain.	[75]
*Ae. aegypti*	Lambda-cyhalothrin	Adult	Genera of *Coprococcus*, *Ruminococcus*, *Bilophila*, *Enterobacter*, *Porphyromonas*, *Bifidobacterium*, *Weissella*, and *Delftia* were enriched in the resistant group. Bacteria *Bacteroides faecichinchillae* decreased significantly in resistant midguts.	[47]
*Ae. aegypti*	Lambda-cyhalothrin	Adult	The presence of *Pseudomonas viridiflava* is associated with pyrethroid degradation. *Parabacteroides*, *Megasphaera*, *Akkermansia*, *Lardizabala*, *Ruminococcus*, and *Coprococcus* genera were enriched in susceptible mosquitoes.	[163]
*Ae. aegypti*	Permethrin, deltamethrin exposure	Adult	After exposure to permethrin, the most abundant bacterial species were *Pantoea agglomerans* and *Pseudomonas azotoformans-fluorescens-synxantha*. *Elizabethkingia meningoseptica* and *Ps. azotoformans-fluorescens-synxantha* were the most abundant after exposure to deltamethrin.	[73]
*Ae. albopictus*	Deltamethrin	Adult	Abundance of *Serratia oryzae* was significantly higher in the resistant strain.	[165]
*Ae. albopictus*	Deltamethrin	Adult	*Acinetobacter junii* and *Se. oryzae* significantly increased after deltamethrin treatment.	[164]
*Ae. stimulans*	Methoprene	Larval	Increased abundances of *Clostridium* spp. and *Lysinibacillus* spp.	[169]
*An. albimanus*	Fenitrothion	Adult	Resistance selection enriches bacterial taxa, reduces diversity, and significantly increases *Bacillus* and *Klebsiella pneumoniae.*	[69]
*An. albimanus*	Alphacypermethrin or permethrin	Adult	The abundance of *Pseudomonas fragi* and *Pa. agglomerans* increased with pyrethroid exposure.	[166]
*An. arabiensis*	Deltamethrin and malathion	Adult	Susceptible mosquitoes showed greater gut bacterial diversity than resistant mosquitoes.	[93]
*An. coluzzii*	Deltamethrin	Adult	*Ochrobactrum*, *Lysinibacillus*, and *Stenotrophomonas* genera were significantly enriched in resistant mosquitoes; *Asaia* and *Serratia* dominated the susceptible individuals.	[150]
*An. gambiae s.s.*	Permethrin	Adult	*Sphingobacterium*, *Lysinibacillus*, *Streptococcus*, and *Rubrobacter* were associated with resistant mosquitoes; *Myxococcus* was associated with susceptible mosquitoes.	[149]
*An. stephensi*	Temephos selection RR > 10	Larval	Resistant strain with 4 dominant genera, i.e., *Pseudomonas*, *Aeromonas*, *Exiguobacterium*, and *Microbacterium*	[28]
*Cx. pipiens*	Organophosphate	Adult	Resistant mosquitoes with higher loads of *Wolbachia*.	[167]
*Cx. pipiens pallens*	*Bti* exposure	Larval	The predominant bacteria changed from *Actinobacteria* to *Firmicutes*, and the abundance of *Actinobacteria* was gradually reduced with an increase in the concentration of *Bti*. At the genus level, *Bacillus* replaced *Microbacterium* as the predominant genus.	[168]
*Cx. quinquefasciatus*	Deltamethrin	Adult	At the genus level, *Aeromonas*, *Morganella*, *Elizabethkingia*, *Enterobacter*, *Cedecea*, and *Thorsellia* showed significant differences between strains. At the species level, *Bacillus cereus*, *Enterobacter cloacae* s.l., *Streptomyces* sp., *Pseudomonas* sp., and *Wolbachia* were more abundant in the resistant strains.	[74]

*Bti*: *Bacillus thuringiensis* var. *israelensis*; *s.s.*: sensu stricto; s.l.: sensu lato.

**Table 2:** Impact of microbiota on insecticide resistance.

Mosquito Species	Insecticide	Mosquito Stage	Description of Findings	Reference
*Ae. aegypti*	Bifenthrin, *Bti*, temephos, methoprene	Adult, larval	Infection with *Wolbachia* has no effect on susceptibility to insecticides.	[170]
*Ae. aegypti*	Propoxur, naled	Larval	Broad-spectrum antibiotic treatment of larvae decreases the metabolic detoxification of propoxur and naled. Adding cultured gut bacteria isolated from mosquito larvae reduces larval mortality.	[177]
*Ae. albopictus*	Deltamethrin	Adult	Cultured bacteria *Serratia oryzae* and *Acinetobacter junii* promote resistance.	[164]
*Ae. albopictus*	Deltamethrin	Adult	The survival of *Se. oryzae*-enriched mosquitoes significantly increased. Three metabolic detoxification enzymes in *Se. oryzae*-enriched mosquitoes increased. Carboxylesterase activity was detected in *Se. oryzae*. *Se. oryzae* can degrade deltamethrin in vitro; degradation efficiency was positively correlated with time and bacterial amount.	[165]
*An. arabiensis*	Deltamethrin, malathion	Adult	Resistance mosquitoes have lower gut bacterial diversity. Supplementation of bacterial *St. pyrogenes* or *Escherichia coli* increased insecticide tolerance. Antibiotic supplementation via sugar decreased tolerance to the insecticides deltamethrin and malathion. Both R/S females had decreased α-esterase activity after gentamicin, streptomycin, vancomycin, heat-killed *St. pyrogenes*, or live *E. coli* treatment. GST, P450, and β-esterase changes are inconsistent.	[93]
*An. gambiae s.s.*	Pyrethroid	Adult	Resistance leads to increased mortality by the fungi *Beauveria bassiana* and *Metarhizium anisopliae* infection.	[175]
*An. gambiae s.s.*, *An. funestus*, *An. arabiensis*	Pyrethroids, organochlorines, carbamates	Adult	Resistant mosquitoes preinfected with *Be. bassiana* or *Me. anisopliae* showed a significant increase in mortality after insecticide exposure.	[174]
*An. stephensi*	Bt	Larval	Commensal microbes in the midgut are capable of degrading insecticidal *Bt* proteins, decreasing larval susceptibility to *Bt*. Antibiotic treatment increased mortality and reached 100% mortality at a concentration of 110 μg/mL of an antibiotic mixture of penicillin, streptomycin, and erythromycin.	[176]
*An. stephensi*	Temephos selection RR > 10	Larval	Adding symbiotic bacteria collected from the breeding place can boost the activity of α-esterase and GST enzymes.	[28]
*Cx. pipiens*	Chlorpyrifos, propoxur	Larval	Infection with *Wolbachia* has no effect on resistance to chlorpyrifos and propoxur.	[171]
*Cx. quinquefasciatus*	DDT	Adult	Infection with *Wolbachia* increases susceptibility to DDT.	[172]
*Cx. quinquefasciatus*	Chlorpyrifos selection	Larval	Activities of acetylcholinesterase (AChE), glutathione S-transferase (GST), esterase (EST), acid phosphatases (ACP), and alkaline phosphatases (ALP) increased in the chlorpyrifos-selected (Chlor-SEL) population. Activities of all enzymes were suppressed when exposed to *Me. anisopliae* or *Be. Bassiana*.	[173]

**Table 3:** Members of the mosquito microbiota that degrade insecticide resistance.

Mosquito Species	Insecticide	Insecticide Degradation Symbionts	Reference
*Ae. aegypti*	Lambda-cyhalothrin	*Pseudomonas viridiflava*	[163]
*An. albimanus*	Fenitrothion	† *Bacillus cereus* and *Acinetobacter baumannii*	[69]
*An. albimanus*	Permethrin, Alphacypermethrin	*Pantoea agglomerans*	[166]
*An. coluzzii*	Deltamethrin	*Ochrobactrum*, *Lysinibacillus*, and *Stenotrophomonas* genera	[150]
*An. gambiae*	Permethrin	*Sphingobacterium*, *Lysinibacillus*, and *Streptococcus* genera	[149]
*An. stephensi*	Temephos	*Pseudomonas* sp., *Aeromonas* sp., *Exiguobacterium* sp., and *Microbacterium* sp.	[28]
Multiple species	Pyrethroids	Bacteria and fungi: *Bacillus* spp., *Raoultella ornithinolytica*, *Pseudomonas fluorescens*, *Brevibacterium* sp., *Acinetobacter* sp., *Aspergillus* sp., *Candida* sp., *Trichoderma* sp., and *Candia* spp.	[181]

† Identified and insecticide degradation function confirmed by the corresponding study.

## Data Availability

All data generated or analyzed during this study are included in this published article and its additional files.
